# Tanzanian Population's Perspective on Facial Profile Esthetic Preferences

**DOI:** 10.1155/ijod/2937725

**Published:** 2025-05-15

**Authors:** Ali Khamis Hamad, Ferdinand Mabula Machibya, Matilda M. Mlangwa, David N. Ngassapa

**Affiliations:** ^1^Department of Anatomy, School of Biomedical Sciences, Muhimbili University of Health and Allied Sciences, Dar es Salaam, Tanzania; ^2^Department of Orthodontics, Paedodontics and Community Dentistry, Muhimbili University of Health and Allied Sciences, Dar es Salaam, Tanzania

## Abstract

**Background:** The perception of facial profile attractiveness varies among individuals and may influence clinical decision-making in orthodontic dentofacial treatment goals.

**Objective:** The aim of the study was to evaluate the facial profile preferences of Tanzanian individuals.

**Materials and Methods:** This cross-sectional study was conducted at Muhimbili University Dental Clinic in Dar es Salaam, Tanzania. A total of 387 participants, including 146 females and 241 males, were selected based on their lack of technical awareness of the facial profiles. The mean age of the male and female participants was 27.94 ± 7.67 and 28.98 ± 7.76 years, respectively. The participants were asked to evaluate male and female facial profiles and score them on a scale of 1–4, with no score being assigned to more than one profile. The relative frequency of the profile scores was calculated, and a *t*-test and analysis of variance (ANOVA) were used to compare the preferences across different groups. A statistical significance was set at a 95% confidence interval (CI), with a *p*-value of *p* < 0.05 considered significant.

**Results:** Among the male profiles, 48.3% (187) of the participants ranked profile M1, “normal maxilla, retruded mandible”, as the most attractive, while 48.1% (186) ranked profile M2, “retruded maxilla, protruded mandible”, as the least preferred. Statistically significant differences were observed between age groups and income groups (*p* < 0.05) in the ratings of each male profile. For the female profiles, 56.6% (219) of the raters preferred profile F4, “straight”, while profile F3, “protruded maxilla, normal mandible”, was ranked as the least preferred by 51.7% (200) of the participants. Significant differences were observed in the ratings of profile F4, “straight”, between groups (*p* < 0.05), except when comparing ratings between genders, where no significant difference was found. No statistically significant differences on age or income were observed for profile F3, “protruded maxilla, normal mandible”.

**Conclusions:** The most preferred profiles among the Tanzanian population were the male profile with a mandibular retrusion (M1) and the female straight profile (F4).

## 1. Background

Over the past few years, research on facial attraction has become a prominent topic in various fields of medical and social sciences, including orthodontics, maxillofacial surgery, plastic surgery, and psychology [1]. One important factor influencing facial attractiveness is age, with studies indicating that younger individuals tend to rate faces as more attractive due to preferences for facial features associated with youth [2]. Similarly, socioeconomic status (SES) has been linked to perceptions of facial attractiveness, with higher SES individuals often associating more prominent or symmetrical features with attractiveness, likely influenced by cultural and social factors [3]. Gender differences also shape attractiveness judgments, with women generally prioritizing facial features that signal health and fertility, while men may focus on features that suggest dominance and strength [4]. Finally, skin color and ethnicity significantly affect attractiveness ratings, as cultural norms and racial biases often influence the esthetic preferences of different populations. For instance, studies show that individuals from African or African-descendant communities tend to rate profiles with features associated with their ethnic group as more attractive [5]. This suggests that ethnic background plays a crucial role in shaping perceptions of facial beauty, highlighting the importance of considering cultural context in attractiveness research.

Although people typically do not view themselves from a profile perspective, previous reports have shown differences in the perceptions of facial profile esthetics between dental practitioners and patients [6], ethnic groups [7], and even genders [8]. For example, in the South African sample, there were no differences between male and female responses, with individuals possessing protrusive profiles rated as more attractive than those with other profile types among Black individuals [9]. In contrast, the facial profile of females with Class I skeletal relationships, particularly among white subjects, was perceived as the most attractive [6]. Additionally, in the 1970s, Foster stated that the fuller lip profile was ranked as the most attractive for youngsters, and was more favored by females than males [10]. In general, the concept of beauty is subjective [11, 12]; consequently, it is often difficult to define treatment goals based on esthetics, as no single profile type would be considered the most attractive by everyone [8]. Occasionally, the patients' opinions on esthetics are overlooked by both general practitioners and dental specialists, which can lead to disappointments with treatment outcomes, as the patient's perception of esthetics may differ from that of the clinicians [13].

Thus, the primary aim of this study was to evaluate the facial profile preferences of Tanzanian individuals. The secondary aims were to assess the influence of gender, age, income, and skin color (ethnicity) on facial profile preferences. The null hypotheses tested in this study were that there is no significant difference in facial profile preferences based on gender, age, income, and skin color.

## 2. Materials and Methods

This cross-sectional study involved 387 participants (241 males and 146 females) of both adolescent and adult age groups, selected based on their lack of technical knowledge of facial profiles. The study was conducted at the Muhimbili University Dental Clinic in Dar es Salaam, Tanzania. The mean age of the male participants was 27.94 ± 7.67 years, while the mean age of the female participants was 28.98 ± 7.76 years. The study was approved by the Muhimbili University Senate Research and Publications Committee (MUHAS-REC-05-2023-1654). Two Tanzanian black models, one male and one female, both with skeletal and dental Class I relationships (confirmed by Steiner's cephalometric tracing) [14], were selected for the preferred profile evaluations. The ages of the models ranged from 22 to 27 years. The participants gave consent for the use of their photographs and lateral cephalograms through signed informed consent forms. Although the original profiles of the models did not exactly match Steiner's S-line, due to the difficulty in finding a model with upper and lower lips precisely touching the S-line (drawn from the soft tissue pogonion to the midpoint of the columella of the nose), they were still very close. Both models displayed slight bimaxillary protrusion.

A digital camera (Lumix, Panasonic Corporation, China) was used to take colored profile images, positioning the participant five feet away from the camera [15] with their heads in a natural position. The lateral cephalograms of the male and female models were taken by the same trained technician using the same cone beam computed tomography (CBCT) (X-VIEW 3D PAN CEPH, Trident S.r.l, Italy) following standard radiation regulations [16]. The images, photographs, and lateral cephalogram were taken at a Dental Radiology unit of the School of Dentistry and then transferred to a Macintosh computer, Version 14.4 Beta (Apple Inc., California, US).

**Figure 1:**
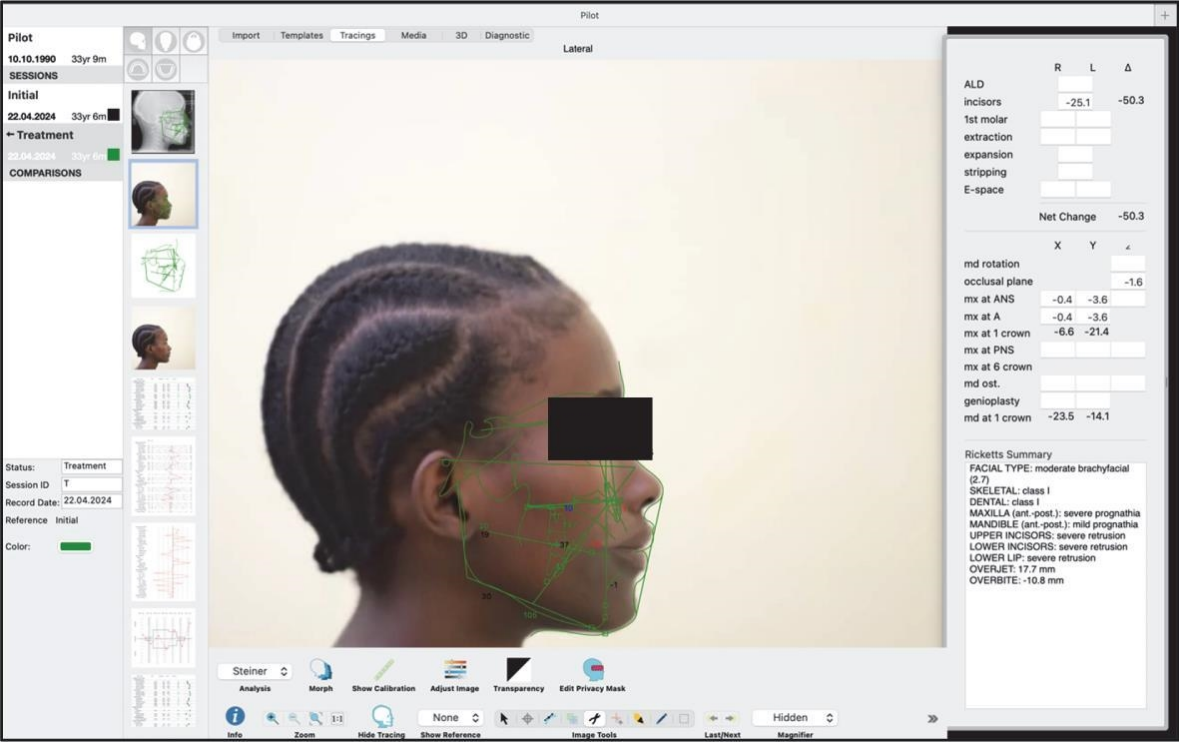
Quick Ceph Studio interface used for generating profile distortions.

For tracing the lateral cephalograms and generating facial profile distortions, Quick Ceph Studio, Version 5.2.6 (Quick Ceph Systems, Inc., FL 34236 US) was used (Figure 1). The initial tracing included three reference lines: the S-N line, a vertical line perpendicular to the S-N line, and the S-line. Based on these tracings, three additional tracings were to simulate changes in the facial profile by moving the maxillary or mandibular alveolar portion either −3 mm backward (retrusion) or 3 mm forward (protrusion) from a vertical line. The modified tracings were as follows: retruded mandible (0/−3): backward positioning of the mandible from a vertical line; retruded maxilla and protruded mandible (−3/+3): backward positioning of the maxilla and forward positioning of the mandible; protruded maxilla and retruded mandible (+3/0): forward positioning of the maxilla and normal positioning of the mandible. The original photographs were digitally manipulated using the same software, generating four images for each gender. These modifications were applied to the original photographs, and the tracing layers were removed before saving the modified images in TIFF format. The images were then sorted by gender and labeled as follows:

Male:

1. M1 = normal maxilla and retruded mandible.
2. M2 = retruded maxilla and protruded mandible.
3. M3 = protruded maxilla and normal mandible.
4. M4 = straight.

Female:

1. F1 = normal maxilla and retruded mandible.
2. F2 = retruded maxilla and protruded mandible.
3. F3 = protruded maxilla and normal mandible.
4. F4 = straight.

**Figure 2:**
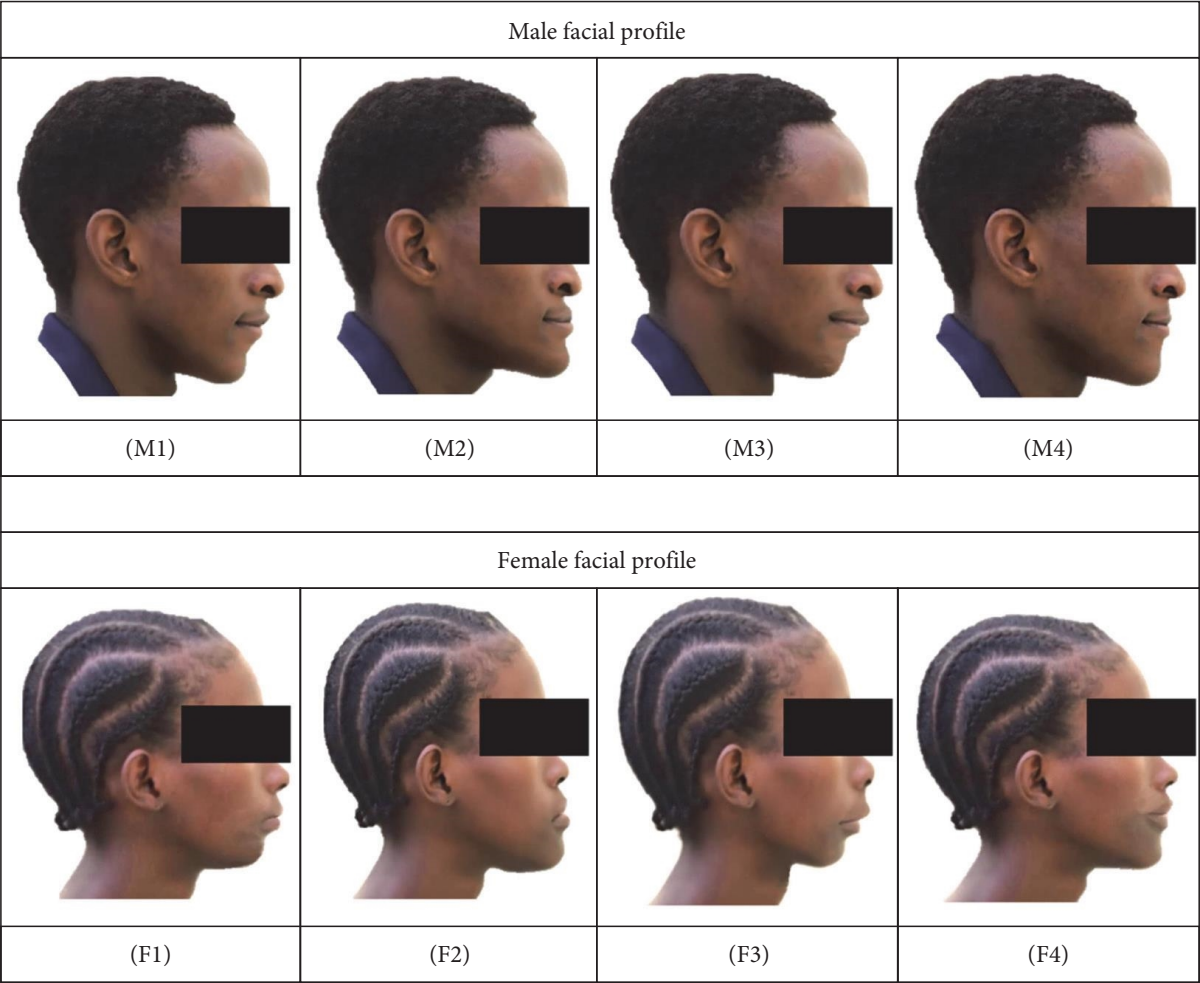
Facial profile distortions. (M1) Normal maxilla with retruded mandible, (M2) retruded maxilla and protruded mandible, (M3) protruded maxilla and normal mandible, (M4) straight, (F1) retruded mandible, (F2) retruded maxilla with protruded mandible, (F3) protruded maxilla with normal mandible, and (F4) straight.

A questionnaire was distributed to gather demographic information from the raters, who evaluated the modified profiles. The photographs (Figure 2) were presented to participants one at a time in printed form, shown in a randomized order to prevent potential bias from evaluating them in a fixed sequence. Each participant was asked to assess and score each image (Figure 2), with scores ranging from 1 to 4, where 1 represented the least attractive, while 4 represented the most attractive profile. They were instructed not to assign the same score to more than one profile. Each participant had 5 min to evaluate and score the four photographs, which were shown in random order for each gender. This time frame was determined based on pretest evaluations to ensure participants had sufficient time to assess each image. Before the evaluation began, participants received clear instructions on how to view and score the photographs. During the evaluation, minimal guidance was provided to avoid bias. Participants were allowed to ask for clarification, though such instances were rare and did not affect the overall evaluation. The evaluation took place in a quiet, controlled environment to minimize distractions, ensuring that no one evaluator influenced another. Demographic details such as gender, age, income, and skin color of the participants were recorded. The age of the raters was categorized as adolescent (under 20 years) and adult (20 years and older). Income was grouped into low (below 300,000 Tanzanian shillings), middle (300,000–1,000,000 Tanzanian shillings), and high (above 1,000,000 Tanzanian shillings). Skin color was categorized into three groups: light, brown, and dark, based on a combination of visual assessment and standard reference guides, including the Fitzpatrick Skin Type Scale. This scale classifies skin tones according to their response to ultraviolet (UV) exposure, ranging from very light (Type I) to very dark (Type VI). In our study, it served as a general guide for classifying participants into the three categories. The assessment was conducted through subjective visual evaluation by a single trained investigator. To ensure consistency, the investigator used a standardized reference chart for skin color and performed all evaluations under similar conditions, specifically, in natural daylight with minimal artificial lighting, to minimize variation and enhance uniformity in classification. Participants who were dental professionals or students were excluded from the study, as were those who chose not to complete the questionnaire. After the 387 participants completed the survey, the data were entered into an RStudio Desktop for macOS 12+, version 2023.12.1 + 402 (Posit Software, BOSTON, US) for analysis. A one-sample Kolmogorov–Smirnov test was performed to assess the normality of the data, yielding a result of *D* = 0.028, *p*-value = 0.915, confirming that the data met the assumptions for parametric statistical analyses. Descriptive statistics, relative frequency distributions, and intraclass correlation coefficients (ICCs) were calculated to assess intrarater agreement for each facial profile. To evaluate reliability, 99 subjects from the pilot study were asked to rank the facial profiles again after a 6-week interval. The ICC was then used to measure the consistency of their responses across the two time points.

The association of the rater responses was compared according to genders, age, color of skin, and income. The *t*-test was used to compare gender and age differences, while the analysis of variance (ANOVA) was applied to compare responses based on personal income and skin color, and across the facial groups. Additionally, post hoc pairwise comparisons were conducted using Tukey's Honest Significant Difference (HSD) test to examine which specific group pairs were significantly different following the significant ANOVA results.

## 3. Results

The distribution of the raters according to demographic information is shown in Table 1. The relative frequency distributions of the participants' preferences for the female and male profiles are presented in Table 2. Among the four ranked male profiles, the majority of participants, 48.3% (187) ranked the male profile with a retruded mandible (M1) as the most attractive and preferred, while the male profile with a retruded maxilla (M2) was ranked as the least preferred by 48.1% (186) of the participants. A statistically significant difference was observed in the ranking of M1 and M2 (*p* < 0.05). For the female profiles, 56.6% (219) of the raters clearly preferred the straight profile (F4), while the profile with a protruded maxilla (F3) was ranked as the least preferred by 51.7% (200) of participants. A statistically significant difference was found in the ranking of F4 (*p* < 0.05), though no significant differences were observed in the ranking of the other female profiles. Regarding reliability, 25% (99 subjects) of the sample size involved in the pilot study were asked to rank the facial profiles again 6 weeks later. The ICC was used to evaluate the intrarater agreement for each facial profile. As shown in Table 3, the test demonstrated excellent reliability for all profiles (*p* < 0.001).

Post-hoc comparisons for the male profile (M1) indicated that the adult group was significantly different from the adolescent group (*p* ~ 0.001). Furthermore, the middle-income group was significantly different from the lower-income group (*p*=0.002), and the high-income group was significantly different from the lower-income group (*p* ~ 0.001). However, no significant differences were found between the middle-income and high-income groups (*p*=0.373). For the female profile (F4), post hoc comparisons showed that the adult group was significantly different from the adolescent group (*p*=0.003). Additionally, the middle-income group was significantly different from the lower-income group (*p*=0.002), and the high-income group was significantly different from the lower-income group (*p* ~ 0.001). No significant differences were observed between the middle-income and high-income groups (*p*=0.818).

### 3.1. The Participants' Preference by Gender

The results indicated that both genders showed a clear preference for the male profile with a retruded mandible (M1) and the female straight profile (F4). The male profile with a retruded maxilla and protrusive mandible (M2), as well as the female profile with a protrusive maxilla (F3), were ranked as the least preferred profiles by both genders. No statistically significant gender differences were observed in the rating of any of the profiles (Tables 4 and 5).

### 3.2. Participants' Preferences by Age

The M4 and F4 profiles were the most preferred among adolescents, while the M2 and F3 profiles were rated as the least preferred (Tables 6 and 7). In contrast, adults preferred the M1 and F4 profiles as the most attractive (Table 6). Statistically significant differences were observed between the adolescent and adult groups in terms of male profile preferences (*p* < 0.05). The adult group preferred the M1 male profile and the F4 female profile (*p* < 0.05), and assigned lower scores to the M2 and F2 profiles compared to the adolescent group.

### 3.3. Participants Preferences by Income

All groups rated profile M1 as the most preferred male profile and profile M2 as the least preferred male profile. The differences in preferences for both the most and least preferred profiles were statistically significant (*p* < 0.05) (Table 8). Furthermore, all groups agreed that the female profile F4 was the most preferred, while profile F3 was the least preferred, with statistically significant differences observed (*p* < 0.05) (Table 9).

### 3.4. Participants Preferences by Their Skin Color

The profile preferences based on the raters' skin color are shown in Tables 10 and 11. In all groups, the male profile with a retruded mandible (M1) and the female straight profile (F4) were the most preferred. The M2 and F3 profiles were rated as the least preferred by all groups. No statistically significant differences were observed between the groups in the ranking of either the male or female profiles.

## 4. Discussion

Various approaches have been used in different studies to determine the facial profile preference of specific populations. In the present study, we used colored profile photographs instead of solid black silhouettes to assess the preferred facial profile of the Tanzanian population. It is important to note that colored profiles provide a more realistic representation of facial esthetics than silhouettes [17]. Our primary aim was to evaluate the opinions of Tanzanian individuals who participated in the study and compare their facial esthetic preferences in relation to their gender, age, income, and skin color variations.

In this study, the male profile with a retruded mandible (M1) emerged as the most preferred profile, followed by the straight profile (M4) as the second most preferred. This finding contrasts with several other studies where the straight profile is often considered the most attractive, particularly in Western populations, where facial symmetry and harmony are regarded as key esthetic qualities [18]. For instance, studies by Brown et al. [19] and Davis et al. [20] found that straight or near-straight male profiles are often rated as the most attractive due to the perceived balance and proportionality of facial features. However, the present study found that the M1 profile, characterized by a retruded mandible, was ranked as the most preferred. This could be attributed to various factors, including cultural differences in esthetic preferences and the specific sample population [21]. Cultural or regional factors might influence preferences for profiles with different jawline characteristics. For example, in some populations, a more pronounced or softer jawline might be considered more masculine or appealing, leading to a preference for profiles that emphasize these traits [22]. Additionally, while the straight profile (M4) was the second most preferred in this study, it was not ranked as the most preferred, as has often been the case in other studies [23]. This discrepancy highlights the variability in esthetic preferences, which can be influenced by factors such as age, gender, and individual differences in esthetic sensibility [24]. The sample in the present study may have been influenced by specific demographic or cultural traits that differ from those in other studies, which may explain why the straight profile was ranked second rather than first [25].

It is important to note that profile preferences, particularly for male faces, are subjective and can vary greatly depending on the population studied. Factors such as cultural norms, historical beauty standards, and contemporary media representations can all influence these preferences [26]. Therefore, while our study shows a preference for the M1 profile with a retruded mandible, this result may not be directly comparable to those from studies conducted in different cultural contexts or using different methodologies. Future studies should explore the underlying reasons for these differences in profile preferences, such as conducting cross-cultural comparisons or exploring the influence of media and societal trends on facial attractiveness [27].

Regarding female profiles, the straight profile (F4) was rated as the most attractive, followed by the F3 profile (with a retruded mandible) as the second most preferred. The F3 profile (protruded maxilla with normal mandible) was rated as the least preferred. This finding aligns with the study conducted in Israel [28] and India [29], where the straight female profile and Class I male profile were most preferred. Similar results were reported in studies involving Chinese [6], Turkish [30], and Indian [31] participants. In contrast, previous literature suggested that a prognathic mandible is a more masculine trait, and a retrognathic profile is considered more feminine [32, 33].

Regarding the least attractive profiles, participants gave the lowest scores to the M2 (most concave male profile) and F3 (a most convex facial profile). This aligns with findings from a study conducted in Iran, where profiles with a protruded mandible were also ranked as the least attractive for both males and females [13]. Notably, profiles with a protruded mandible received lower score compared to those with a protruded maxilla, particularly in female profiles. This indicates that the maxillary position was considered more crucial for both groups. In contrast to a study conducted among the Chinese population [34], which emphasized the importance of the mandibular position in facial profiles assessments [6], our findings suggest that the protruded maxilla, with a normal mandibular profile, was rated as the least preferred female facial profile.

Conflicting findings have emerged from studies examining the influence of demographic characteristics on facial esthetic preferences [35]. Many earlier studies found no significant differences in facial esthetic assessments based on gender, age, income, and skin color. Similarly, our study showed no statistically significant relationship between these demographic factors and esthetic preferences, except for the age of the participant. Our findings differ slightly from those of the previous study, where children's preferences regarding facial attractiveness were similar to those of adults [36, 37]. In this study, many adults preferred the M1 profile (with a retruded mandible), while adolescents preferred the M4 profile (with a straight profile). These findings suggest that preferences may shift with age, from a preference for straight profiles to a preference for retruded mandibular profiles. Our findings also align with a study conducted in Turkish [30]. Conversely, a study by Cochrane et al. [38] reported that the majority of females preferred orthognathic profiles compared to their male counterparts. Previous studies also noted that female patients with a positive orthodontic history tended to give higher ratings to straight profiles in males and bimaxillary protrusion profiles in females [39].

Regarding social income, statistically significant differences were observed in the evaluation of profile preferences. The profiles most preferred by participants were more accepted among individuals with middle and high income, while no noticeable differences were found within the lower income group. This suggests that individuals with high income may be more concerned about their facial profiles and more likely to seek orthodontic treatment to improve them compared to those with lower income.

In this study, skin color did not influence facial profile preferences, as all participants, regardless of skin color, preferred male profiles with a retruded mandible (M1) and female profiles with a straight profile (F4). This contrasts with findings from studies conducted in other countries. For example, Beukes [40] found that black male participants preferred slightly convex profiles, while straight profiles were preferred by individuals with white skin. Similarly, Salinas-Mendoza [41] reported that black males favored more convex profiles, while individuals with white skin preferred straighter profiles. These studies suggest that skin color may influence the perception of facial esthetics, with different cultural groups placing varying degrees of importance on specific profile features. However, the divergence between our study's findings and those from other countries may be attributed to the distinct cultural contexts in which these preferences were formed. In the Tanzanian community, the preference for a retruded mandible in male profiles (M1) and a straight profile (F4) in females may be influenced by specific ethnic norms, historical influences, and local media representations, which differ from those seen in Western or other global populations.

While our study found no difference in preferences based on skin color, it is important to understand how cultural variations shape perceptions of beauty in different populations. For instance, individuals with lighter skin tones may place a higher value on symmetrical, straight profiles due to historical European beauty standards, which emphasize symmetry and proportionality [42]. In contrast, individuals with black or brown skin tones may have different associations with facial features, where softer, more pronounced jawlines or slightly convex profiles may be viewed as more desirable or masculine [43]. Given the diversity in esthetic preferences across populations, further research on the intersection of skin color and profile preferences is essential. Cross-cultural studies, particularly those focusing on East African populations like the Tanzanian community, could provide valuable insights into how cultural, historical, and media influences shape facial attractiveness preferences.

The noticeable discrepancies in sample size between gender and age groups may have influenced the interpretation and generalizability of the study findings. In this study, males comprised 62.27% of the sample, while females represented only 37.73%. Similarly, adults made up a significant majority (81.14%) compared to adolescents (18.86%). These imbalances may have introduced bias in the analysis of esthetic preferences, potentially skewing the results toward the dominant groups. For example, if males or adults have systematically different preferences from females or adolescents, the overall trends identified in the study may not accurately reflect the views of the underrepresented groups. Consequently, the results should be interpreted with caution, especially when drawing conclusions about gender- or age-specific preferences. Future studies should aim for more balanced representation across gender and age categories to enhance the reliability and generalizability of the findings across diverse demographic groups.

Finally, it is crucial to emphasize that these findings should not be translated into specific orthodontic treatment guidelines. Our study was not designed to assess the functional or health-related consequences of different occlusal relationships, nor was it intended to replace established orthodontic treatment goals, which prioritize functional occlusion, dental health, and overall facial harmony. The universal goal of orthodontic treatment—achieving a Class I occlusion—is supported by extensive evidence demonstrating its benefits for both functional and esthetic outcomes. It is important to balance esthetic preferences with functional considerations when planning treatment. Additionally, the findings of our study do not suggest that functional appliance therapy should be avoided for the Tanzanian community. Functional appliance therapy is necessary to address malocclusion that can affect chewing, speech, and long-term dental health. The esthetic preferences identified in our study should not override the functional objectives of orthodontic care, and treatment decisions should be based on clinical needs and long-term outcomes rather than solely on perceived esthetics. Lastly, the results of this study should not be interpreted as an endorsement of orthognathic surgery to achieve a Class II retrusive mandible in males. Orthognathic surgery aims to improve both function and esthetics based on the individual patient's needs, with the goal of achieving an optimal, balanced facial profile and occlusion tailored to the patient's specific condition, rather than adhering strictly to a preference for a retruded mandible.

## 5. Study Limitations

One limitation of this study is the method used to categorize skin color, which was based on subjective visual assessment rather than objective measurement tools. Although a standardized reference chart and consistent natural lighting conditions were used to improve uniformity, the classification remains susceptible to observer bias and potential inconsistencies. Future studies may benefit from using more objective techniques, such as spectrophotometry or digital image analysis, to enhance accuracy and reproducibility.

Additionally, the data were limited by the specific ethnic backgrounds and range of facial types included in the study. All participants were from the Tanzanian population, and facial profile preference may vary among different ethnic groups. Moreover, the maxillary and mandibular alveolar segments were only adjusted in increments or decrements of 3 mm from their original position. In reality, patients often present with more complex facial variations. Therefore, future research should consider a broader range of ethnicities and more diverse facial types to improve generalizability.

## 6. Conclusion

The most preferred facial profiles were M1 (normal maxilla, retruded mandible) for males and F4 (straight) for females. In contrast, the least preferred profiles were M2 (retruded maxilla, protruded mandible) for males and F3 (protruded maxilla, normal mandible) for females. The results of this study also indicate that while age and income were significant factors, gender was not found to have a statistically significant influence on facial profile preferences.

## Figures and Tables

**Table 1:** Demographic information of participants.

Variable	Category	*N*	Percentage (%)
Gender	Males	241	62.27
	Females	146	37.73

Age	Adolescents	73	18.86
	Adults	314	81.14

Income	Low	167	43.15
	Middle	132	34.11
	Higher	88	22.74

Skin Color	Black	149	38.50
	White	46	11.89
	Brown	192	49.61

*Note*: This table presents the frequency (*N*) and percentage (%) for each demographic category.

**Table 2:** Relative frequency distributions of the participants' preferences of the male and female profiles.

Profile	Rank				*F* value	*p*-Value
	1 (*N* = 55, 14.2%)	2 (*N* = 41, 10.6%)	3 (*N* = 104, 26.9%)	4 (*N* = 187, 48.3%)		
M1	55 (14.2%)	41 (10.6%)	104 (26.9%)	187 (48.3%)	9.04	0.003
M2	186 (48.1%)	108 (27.9%)	49 (12.7%)	44 (11.4%)	6.57	0.011
M3	111 (28.7%)	168 (43.4%)	70 (18.1%)	38 (9.8%)	2.08	0.15
M4	35 (9.0%)	70 (18.1%)	163 (42.1%)	119 (30.8%)	0.79	0.376

F1	27 (6.9%)	113 (29.2%)	171 (44.2%)	76 (19.6%)	1.16	0.281
F2	96 (24.7%)	167 (43.2%)	92 (23.8%)	32 (8.3%)	2.96	0.086
F3	200 (51.7%)	83 (21.4%)	44 (11.4%)	60 (15.5%)	3.02	0.083
F4	62 (16.0%)	27 (6.9%)	79 (20.4%)	219 (56.6%)	5.52	0.019

*Note: N* represents the number of participants, % represents the percentage of participants within each rank. The statistical significance was determined using ANOVA, with significance indicated at *p* < 0.05.

**Table 3:** Test-retest reliability (using intraclass correlation).

Profiles	*n*	ICC	Lower bound	Upper bound	*p*-Value
F1	96	0.84	0.77	0.89	~0.001
F2	96	0.86	0.80	0.91	~0.001
F3	96	0.93	0.89	0.95	~0.001
F4	96	0.93	0.89	0.95	~0.001

M1	96	0.84	0.77	0.89	~0.001
M2	96	0.85	0.79	0.90	~0.001
M3	96	0.83	0.76	0.88	~0.001
M4	96	0.84	0.77	0.89	~0.001

*Note*: *n*, number of participants. Significant at *p* < 0.05.

Abbreviation: ICC, intraclass correlation coefficients.

**Table 4:** Relative frequency distributions of the participants' preferences of the male profiles by gender.

Gender	Rank	Profile				
		M1 (*N*, %)	M2 (*N*, %)	M3 (*N*, %)	M4 (*N*, %)	Significance
Males	1	37 (15.4%)	112 (46.5%)	68 (28.2%)	24 (9.9%)	ns
	2	29 (12.0%)	67 (27.8%)	98 (40.7%)	47 (19.5%)	ns
	3	57 (23.7%)	34 (14.1%)	48 (19.9%)	102 (42.3%)	ns
	4	118 (48.9%)	28 (11.6%)	27 (11.2%)	68 (28.2%)	ns

Females	1	18 (12.3%)	74 (50.7%)	43 (29.5%)	11 (7.5%)	ns
	2	12 (8.2%)	41 (28.1%)	70 (47.9%)	23 (15.8%)	ns
	3	47 (32.2%)	15 (10.3%)	22 (15.1%)	61 (41.8%)	ns
	4	69 (47.2%)	16 (10.9%)	21 (7.5%)	51 (34.9%)	ns

*Note: N* represents the number of participants; % represents the percentage of participants within each rank for M1, M2, M3, and M4. Significance: ns = not statistically significant. Statistical analysis was conducted using ANOVA to test for differences across the groups, with significance indicated at *p* < 0.05.

**Table 5:** Comparison of the relative frequency distributions of the participants' preferences of the female profiles by gender.

Gender	Rank	Profile				
		F1 (*N*, %)	F2 (*N*, %)	F3 (*N*, %)	F4 (*N*, %)	Significance
Males	1	14 (5.8%)	58 (24.1%)	126 (52.3%)	41 (17.0%)	ns
	2	76 (31.5%)	101 (41.9%)	50 (20.7%)	17 (7.1%)	ns
	3	99 (41.1%)	63 (26.1%)	25 (10.4%)	53 (22.0%)	ns
	4	52 (21.6%)	19 (7.9%)	20 (16.6%)	130 (53.9%)	ns

Females	1	13 (8.9%)	38 (26.0%)	74 (50.9%)	21 (14.4%)	ns
	2	37 (25.3%)	66 (45.2%)	33 (22.6%)	10 (6.8%)	ns
	3	72 (49.3%)	29 (19.9%)	19 (13.0%)	26 (17.8%)	ns
	4	24 (16.4%)	13 (8.9%)	20 (13.7%)	89 (60.9%)	ns

*Note: N* represents the number of participants; % represents the percentage of participants within each rank for F1, F2, F3, and F4. Significance: ns = not statistically significant. Statistical analysis was conducted using ANOVA to test for differences across the groups, with significance indicated at *p* < 0.05.

**Table 6:** Comparison of the relative frequency distributions of the participants' preferences of the male profile by age.

Age	Rank	Profile				
		M1 (*N*, %)	M2 (*N*, %)	M3 (*N*, %)	M4 (*N*, %)	Significance
Adolescents	1	20 (27.4%)	25 (34.2%)	20 (27.4%)	8 (10.9%)	*s*
	2	10 (13.7%)	24 (32.9%)	24 (32.9%)	15 (20.5%)	*s*
	3	20 (27.4%)	10 (13.7%)	20 (27.4%)	23 (31.5%)	*s*
	4	23 (31.5%)	14 (19.2%)	9 (12.3%)	27 (36.9%)	*s*

Adults	1	35 (11.1%)	161 (51.3%)	91 (28.9%)	27 (8.6%)	ns
	2	31 (9.9%)	84 (26.8%)	144 (45.9%)	55 (17.5%)	ns
	3	84 (26.8%)	39 (12.4%)	50 (15.9%)	140 (44.6%)	ns
	4	164 (52.2%)	30 (9.6%)	29 (9.2%)	92 (29.3%)	ns

*Note*: *N* represents the number of participants; % represents the percentage of participants within each rank for M1, M2, M3, and M4. Significance: *s* = statistically significant; ns = not statistically significant. Statistical analysis was conducted using ANOVA to test for differences across the groups, with significance indicated at *p* < 0.05.

**Table 7:** Comparison of the relative frequency distributions of the participants' preferences of the female profile by age.

Age	Rank	Profile				
		F1 (*N*, %)	F2 (*N*, %)	F3 (*N*, %)	F4 (*N*, %)	Significance
Adolescents	1	8 (10.9%)	14 (19.2%)	31 (42.4%)	19 (26.0%)	ns
	2	24 (32.9%)	27 (36.9%)	14 (19.2%)	9 (12.3%)	ns
	3	26 (35.6%)	21 (28.8%)	15 (20.5%)	11 (15.1%)	ns
	4	15 (20.5%)	11 (15.1%)	13 (17.8%)	34 (46.6%)	ns

Adults	1	19 (6.1%)	82 (26.1%)	169 (53.8%)	43 (13.7%)	*s*
	2	89 (28.3%)	140 (44.6%)	69 (21.9%)	18 (5.7%)	*s*
	3	145 (46.2%)	71 (22.6%)	29 (9.2%)	68 (21.7%)	ns
	4	61 (19.4%)	21 (6.7%)	47 (14.9%)	185 (58.9%)	*s*

*Note: N* represents the number of participants; % represents the percentage of participants within each rank for F1, F2, F3, and F4. Significance: *s* = statistically significant; ns = not statistically significant. Statistical analysis was conducted using ANOVA to test for differences across the groups, with significance indicated at *p* < 0.05.

**Table 8:** Comparison of the relative frequency distributions of the participants' preferences of the male profile by income.

Income	Rank	Profile				
		M1 (*N*, %)	M2 (*N*, %)	M3 (*N*, %)	M4 (*N*, %)	Significance
Low	1	36 (21.6%)	60 (35.9%)	50 (29.9%)	21 (12.6%)	*s*
	2	24 (14.4%)	51 (30.5%)	59 (35.3%)	33 (19.8%)	*s*
	3	42 (25.1%)	31 (18.6%)	35 (20.9%)	59 (35.3%)	ns
	4	65 (38.9%)	25 (14.9%)	23 (13.8%)	54 (32.3%)	ns

Middle	1	13 (9.8%)	77 (58.3%)	35 (26.5%)	7 (5.3%)	*s*
	2	11 (8.3%)	29 (21.9%)	68 (51.5%)	24 (18.2%)	*s*
	3	41 (31.1%)	11 (8.3%)	23 (17.4%)	57 (43.2%)	ns
	4	67 (50.8%)	15 (11.4%)	6 (4.5%)	44 (33.3%)	ns

High	1	6 (6.8%)	49 (55.7%)	26 (29.5%)	7 (7.9%)	*s*
	2	6 (6.8%)	28 (31.8%)	41 (46.6%)	13 (14.8%)	*s*
	3	21 (23.7%)	7 (7.9%)	12 (13.6%)	47 (53.4%)	ns
	4	55 (62.5%)	4 (4.5%)	9 (10.2%)	21 (23.9%)	ns

*Note: N* represents the number of participants; % represents the percentage of participants within each rank for M1, M2, M3, and M4. Significance: *s* = statistically significant; ns = not statistically significant. Statistical analysis was conducted using ANOVA to test for differences across the groups, with significance indicated at *p* < 0.05.

**Table 9:** Comparison of the relative frequency distributions of the participants' preferences of the female profile by income.

Income	Rank	Profile				
		F1 (*N*, %)	F2 (*N*, %)	F3 (*N*, %)	F4 (*N*, %)	Significance
Low	1	14 (8.4%)	37 (22.2%)	74 (44.3%)	41 (24.6%)	ns
	2	52 (31.1%)	71 (42.5%)	34 (20.4%)	12 (7.2%)	ns
	3	64 (38.3%)	44 (26.3%)	23 (13.8%)	35 (20.9%)	*s*
	4	37 (22.2%)	15 (8.9%)	36 (21.6%)	79 (47.3%)	*s*

Middle	1	5 (3.8%)	33 (25.0%)	77 (58.3%)	16 (12.1%)	ns
	2	42 (31.8%)	55 (41.7%)	27 (20.5%)	9 (6.8%)	ns
	3	64 (48.5%)	32 (24.2%)	15 (11.4%)	21 (15.9%)	*s*
	4	21 (15.9%)	12 (9.1%)	13 (9.8%)	86 (65.2%)	*s*

High	1	8 (9.1%)	26 (29.5%)	49 (55.7%)	5 (5.7%)	ns
	2	19 (21.6%)	41 (46.6%)	22 (25.0%)	6 (6.8%)	ns
	3	43 (48.9%)	16 (18.2%)	6 (6.8%)	23 (26.1%)	*s*
	4	18 (20.5%)	5 (5.7%)	11 (12.5%)	54 (61.4%)	*s*

*Note: N* represents the number of participants; % represents the percentage of participants within each rank for F1, F2, F3, and F4. Significance: *s* = statistically significant; ns = not statistically significant. Statistical analysis was conducted using ANOVA to test for differences across the groups, with significance indicated at *p* < 0.05.

**Table 10:** Comparison of the relative frequency distributions of the participants' preferences of the male profile by their skin colors.

Skin color	Rank	Profile				
		M1 (*N*, %)	M2 (*N*, %)	M3 (*N*, %)	M4 (*N*, %)	Significance
Dark	1	21 (14.1%)	67 (44.9%)	44 (29.5%)	17 (11.4%)	ns
	2	16 (10.7%)	48 (32.2%)	59 (39.6%)	26 (17.4%)	ns
	3	38 (25.5%)	18 (12.1%)	28 (18.8%)	65 (43.6%)	ns
	4	74 (49.7%)	16 (10.7%)	18 (12.1%)	41 (27.5%)	ns

Brown	1	8 (17.4%)	24 (52.2%)	12 (26.1%)	2 (4.3%)	ns
	2	3 (6.5%)	10 (21.7%)	23 (50.0%)	10 (21.7%)	ns
	3	16 (34.8%)	6 (13.0%)	7 (15.2%)	17 (36.9%)	ns
	4	19 (41.3%)	6 (13.0%)	4 (8.7%)	17 (36.9%)	ns

Light	1	26 (13.5%)	95 (49.5%)	55 (28.6%)	16 (8.3%)	ns
	2	22 (11.5%)	50 (26.0%)	86 (44.8%)	16 (17.7%)	ns
	3	50 (26.0%)	25 (13.0%)	35 (18.2%)	81 (42.2%)	ns
	4	94 (48.9%)	22 (11.5%)	16 (8.3%)	61 (31.8%)	ns

*Note: N* represents the number of participants in each category; % represents the percentage of participants within each rank for M1, M2, M3, and M4. Significance: ns = not statistically significant; Statistical analysis was conducted using ANOVA to test for differences across the groups, with significance indicated at *p* < 0.05.

**Table 11:** Comparison of the relative frequency distributions of the participants' preferences of the female profile by their skin colors.

Skin color	Rank	Profile				
		F1 (*N*, %)	F2 (*N*, %)	F3 (*N*, %)	F4 (*N*, %)	Significance
Dark	1	10 (6.7%)	40 (26.8%)	75 (50.3%)	24 (16.1%)	ns
	2	41 (27.5%)	63 (42.3%)	38 (25.5%)	7 (4.7%)	ns
	3	71 (47.7%)	36 (24.2%)	13 (8.7%)	29 (19.5%)	ns
	4	27 (18.1%)	10 (6.7%)	23 (15.4%)	89 (59.7%)	ns

Brown	1	6 (13.0%)	13 (28.3%)	19 (41.3%)	8 (17.4%)	ns
	2	14 (30.4%)	14 (30.4%)	14 (30.4%)	5 (10.9%)	ns
	3	18 (39.1%)	17 (36.9%)	3 (6.5%)	7 (15.2%)	ns
	4	8 (17.4%)	2 (4.3%)	10 (21.7%)	26 (56.5%)	ns

Light	1	11 (5.7%)	43 (22.4%)	106 (55.2%)	30 (15.6%)	ns
	2	58 (30.2%)	90 (46.9%)	31 (16.1%)	15 (7.8%)	ns
	3	82 (42.7%)	39 (20.3%)	31 (14.6%)	43 (22.4%)	ns
	4	41 (21.4%)	20 (10.4%)	27 (14.1%)	104 (54.2%)	ns

*Note: N* represents the number of participants in each category; % represents the percentage of participants within each rank for F1, F2, F3, and F4. Significance: ns = not statistically significant. Statistical analysis was conducted using ANOVA to test for differences across the groups. No statistically significant differences were found, with significance indicated at *p* < 0.05.

## Data Availability

The data that support the findings of this study are available from the corresponding author upon reasonable request.
